# Identification of M2 macrophage markers for predicting outcome and therapeutic response in osteosarcoma: Integrated analysis of single-cell and bulk RNA-sequencing

**DOI:** 10.7150/jca.104855

**Published:** 2025-02-28

**Authors:** Yang Liu, Liwei Liu, Xianpeng Wei, Yan Xiong, Qifang Han, Tianhui Gong, Fuzhou Tang, Kaide Xia, Shuguang Zheng

**Affiliations:** 1 Guizhou University of Traditional Chinese Medicine, Guiyang, China.; 2 The First Affiliated Hospital of Guizhou University of Traditional Chinese Medicine, Guiyang, China.; 3 Guiyang Maternal and Child Health Care Hospital, Guiyang Children's Hospital, Guiyang, China.; 4 Guizhou Medical University, Guiyang, China.

**Keywords:** Single-cell and bulk RNA-sequencing, M2 macrophage, prognostic signature, therapeutic response, osteosarcoma

## Abstract

Identification of effective biomarkers is crucial to improve the efficacy of immunotherapy in patients with osteosarcoma. Tumor-associated M2 macrophages, an important immune cell type in the tumor immune microenvironment, are closely related to the formation and progression of tumors. However, the relationships of M2 macrophages and prognosis and the immunotherapy response to osteosarcoma remain unclear. In this study, we obtained single-cell RNA sequencing (scRNA-seq) data of osteosarcoma from the gene expression omnibus (GEO) database and performed trajectory analysis and cell communication analysis. We then identified M2 macrophage marker genes based on scRNA-seq data of osteosarcoma, and constructed a risk-score model using these genes. Next, we compared the survival status and immune features of patients with high and low risk scores. Based on scRNA-seq data, we found that macrophages were the major immune cell type in the osteosarcoma microenvironment, and the high proportion of M2 macrophages might result from the transition of macrophages M1 to M2. M2 macrophages communicated with osteoblastic cells via the APP, MIF, and SPP1 signaling pathways, facilitating osteosarcoma development. Moreover, we identified 189 osteosarcoma-related M2 macrophage marker genes and screened out 10 key genes used for model constrcution. These 10 genes consisted of two known M2 macrophage markers and eight novel M2 macrophage marker genes. Low-risk patients have a statistically significant survival advantage, which was verified in the four GEO datasets. Low-risk patients also displayed a high abundance of tumor-infiltrating immune cells, indicative of an “hot” immune phenotype, while high-risk patients displayed an opposite immunologic feature. Notably, our analysis of two independent immunotherapy cohorts revealed that low-risk patients had good immunotherapy responses and outcomes. Additionally, we determined 32 evidently correlated pairs between risk score and drug sensitivity. This study reveals a new prognostic signature based on M2 macrophage marker genes that can help optimize personalized prognosis and improve immunotherapy outcomes in patients with osteosarcoma and also provides a method for identifying effective biomarkers based on integrated analysis of single-cell and bulk RNA sequencing.

## Introduction

Osteosarcoma, the most common primary malignant bone tumor, is characterized by high incidence rates and poor clinical outcomes 1. An effective biomarker for the prognosis and therapy of osteosarcoma would help to accurately predict the survival of patients, follow disease progression, and develop treatment agents 2. Numerous trials have revealed that multiple drugs, such as chemotherapy agents (doxorubicin and cisplatin), bone-modifying agents (ifosfamide and etoposide), and tyrosine kinase inhibitors (robatumumab and sorafenib), improve the clinical outcomes of osteosarcoma patients, but the long-term survival rate of patients remains low 3. Besides, immunotherapy is a promising strategy that made significant achievements in treating various cancers. In particular, mifamurtide, an immune modulator, has been used successfully to treat patients with osteosarcoma 4. Although major efforts have been made to identify biomarkers allowing to predict immunotherapy response, such as tumor mutation burden and PD-L1 protein expression, the population of patients who benefit from immunotherapy has remained very limited because these biomarkers incompletely reflect the heterogeneous tumor microenvironment (TME) 5. Thus, it is urgent to identify a novel and valuable biomarker for predicting the treatment response and outcome of patients with osteosarcoma.

Cancer cells dwell in a TME composed of different cell types, such as fibroblasts, endothelial cells, and immune cells. Immune cells, one of the key components of the TME, play a crucial role in tumorigenesis and tumor progression. Adaptive immune cells affect not only patients' prognosis but also their responses to immunotherapy. Regarding antitumor immunity, the adaptive T-cell response is a primary focus of research 6, but the role of innate immune cells has not received adequate attention. Macrophages, a critical innate immune cell type, are highly abundant tumor-infiltrating immune cells in osteosarcoma; they can be divided into the immune-stimulatory macrophages (M1 macrophages) and immune-regulatory macrophages (M2 macrophages) subtypes 7. M2 macrophages promote tumor progression by suppressing anti-tumor immunity, which regulates T-cell behavior 8. Immunosuppressive cytokines, (e.g., interleukin 10), generated by M2 macrophages recruit regulatory T cells and inhibit T-cell activation and proliferation, leading to a tolerogenic immune response 9. Remarkably, enhanced M2 macrophages infiltration is linked to poor outcomes across multiple tumor types, including osteosarcoma 10. Nowadays, tumor-associated macrophage-targeted therapy is considered a promising immunotherapeutic strategy against osteosarcoma 11. The anti-inflammatory role of M2 macrophages warrants conducting a comprehensive molecular analysis of M2 macrophages in osteosarcoma.

Single-cell RNA sequencing (scRNA-seq) is widely used to reveal tumor heterogeneity and diversity, raising the possibility of personalized cancer treatments 12. Exploring gene expression signatures based on molecular features of immune cells from scRNA-seq data could be a novel approach to identify functional biomarkers. In this study, we constructed the M2 macrophage landscape in the osteosarcoma microenvironment and performed cell communication analysis to explore the interaction between M2 macrophages and osteoblastic cells. We then identified M2 macrophage marker genes and constructed a risk model for predicting patients' outcomes. Next, we evaluated the predictive power of the signature using multiple independent datasets. Additionally, we explored the relationships between risk scores and TME feature, immunotherapy response, and drug sensitivity.

## Materials and methods

### Data acquisition

We obtained single-cell transcriptomics data of osteosarcoma patients from the GSE152048 dataset of the GEO database (http://www.ncbi.nlm.nih.gov/geo/). We obtained bulk RNA-sequencing data and clinical information on osteosarcoma samples and pan-cancer data from the cancer genome atlas (TCGA) database (https://portal.gdc.cancer.gov). We then downloaded four independent datasets of osteosarcoma samples, namely GSE39058, GSE21257, GSE16091, and GSE39055 from the GEO database for external validation. We also extracted two immunotherapy datasets that was composed of patients undergoing immune checkpoint inhibitor treatment. The immunetherapy patient cohorts, the GSE135222 cohort and IMvigor210 cohort, were respectively collected from the GEO database and http://research-pub.gene.com/IMvigor210CoreBiologies. The Harmony algorithm was been used to eliminate batch effects. For each bulk transcriptomics dataset, samples with incomplete clinical information or follow-up period less than 30 days were removed from the survival analysis. The clinical information of patients from the single-cell dataset is shown in Table S1 and the summary information for all the datasets from the GEO database used in this work is shown in Table S2.

### scRNA-seq analysis

To identify M2 macrophage marker genes, we performed single-cell analysis based on the GSE135222 cohort. For quality control, cells with less than 300 expressed genes, cells with more than 3% mitochondrial genes, and genes expressed in less than three cells were excluded. Next, scRNA-seq data were normalized using the R function “NormalizeData” in the “Seurat” R package, followed by transforming these normalized scRNA-seq data into a Seurat object 13. We then conducted a principal component analysis on the 3000 most variable genes and extracted the first 15 principal components. T-distributed stochastic neighbor embedding (t-SNE) was used to visualize the clustering results for cells 14. Furthermore, we identified differentially expressed genes (DEGs) in each cell cluster using a Wilcoxon test with cutoff threshold values of |log2 (fold change)| > 1 and adjusted* P*-value< 0.05.

### Cell communication analysis

The cell-cell interactions were analyzed by using R package “CellChat” based on the expression of receptors and ligands. The highly expressed co-receptors were identified by using the “identifyOverExpressedGenes” function. We further evaluated coreceptor interactions through using the “identifyOver ExpressedInteractions” function. Finally, we analyzed cellular interactions and aggregated communication networks between cells through using the “ComputeCommunProb” and “computeCommunProb Pathway” functions.

### Establishment and validation of risk model

Based on the bulk RNA-sequencing data and clinical information of osteosarcoma samples from TCGA, we first determined prognosis-related M2 macrophage marker genes using a univariate Cox regression analysis. A least absolute shrinkage and selection operator (LASSO) Cox regression algorithm was then conducted to avoid overfitting. Finally, we included the identified genes in a multiple Cox regression analysis. We built a risk model through a linear combination of mRNA levels of the genes and corresponding risk coefficients. The median value of the risk score was used as a cutoff point to distinguish high- and low-risk score patients. The difference in the overall survival of the two subgroups was assessed using Kaplan-Meier survival analysis. The same approach was performed for validating the outcome prediction ability in four independent cohorts.

### Pathway enrichment analysis

Pathway enrichment analysis was performed using Kyoto encyclopedia of genes and genomes (KEGG) analysis and Gene set enrichment analysis (GSEA) 15. For the KEGG analysis, a *P*-value below 0.05 for the pathway enrichment was considered statistically significant. For GSEA, a |normalized enrichment score (NES)| above 1, a *P*-value below 0.05, and false discovery rate *q*-value below 0.25 were used as the criterion for screening the enriched pathways.

### Analysis of tumor immune microenvironment

We calculated the immune score, stromal score, Estimation of STromal and Immune cells in MAlignant Tumor tissues using expression data (ESTIMATE) score, and tumor purity of high- and low-risk patients by applying the expression data (ESTIMATE) algorithm 16. To evaluate the levels of tumor-infiltrating immune cells in the two risk-score subgroups, we conducted a single sample gene set enrichment analysis (ssGSEA). We also analyzed the levels of immune checkpoint proteins (ICPs), immunogenic cell death (ICD) regulators, and human leukocyte antigen (HLA)-associated genes in the different risk subgroups. We performed a Wilcoxon test with a *P*-value below 0.05 indicating statistical significance.

### Prediction of response to immune checkpoint blockade treatment

Patients undergoing anti-PD-L1 therapy from the IMvigor210 cohort and undergoing anti-PD-1/PD-L1therapy from GSE1335222 were respectively divided into two subgroups, namely the complete response or partial response (RD) subgroup and progressive disease or stable disease (NR) subgroup. The difference in the risk scores of patients between the two subgroups was evaluated using a Wilcoxon test. The difference in the survival of patients between the high- and low-risk subgroups was tested analyzed using a log-rank test. A *p*-value below 0.05 indicated statistical significance.

### Correlation analysis of risk score and drug sensitivity

We obtained the transcription profiles of cancer cell lines, drug response for antitumor drugs in cancer cell lines, and targets/pathways of drugs from Genomics of Drug Sensitivity in Cancer (GDSC) project (http://www.cancerrxgene.org/downloads) 17. We assessed the correlation between risk score and drug sensitivity using Spearman correlation analysis. A false discovery rate below 0.05 indicated a significant correlation.

### Statistical analysis

Statistical analyses were performed by using R software version 4.1.3. A *P*-value below 0.05 was rendered statistically significant.

## Results

### Macrophages are the main immune cell type in the osteosarcoma microenvironment

**Figure 1:**
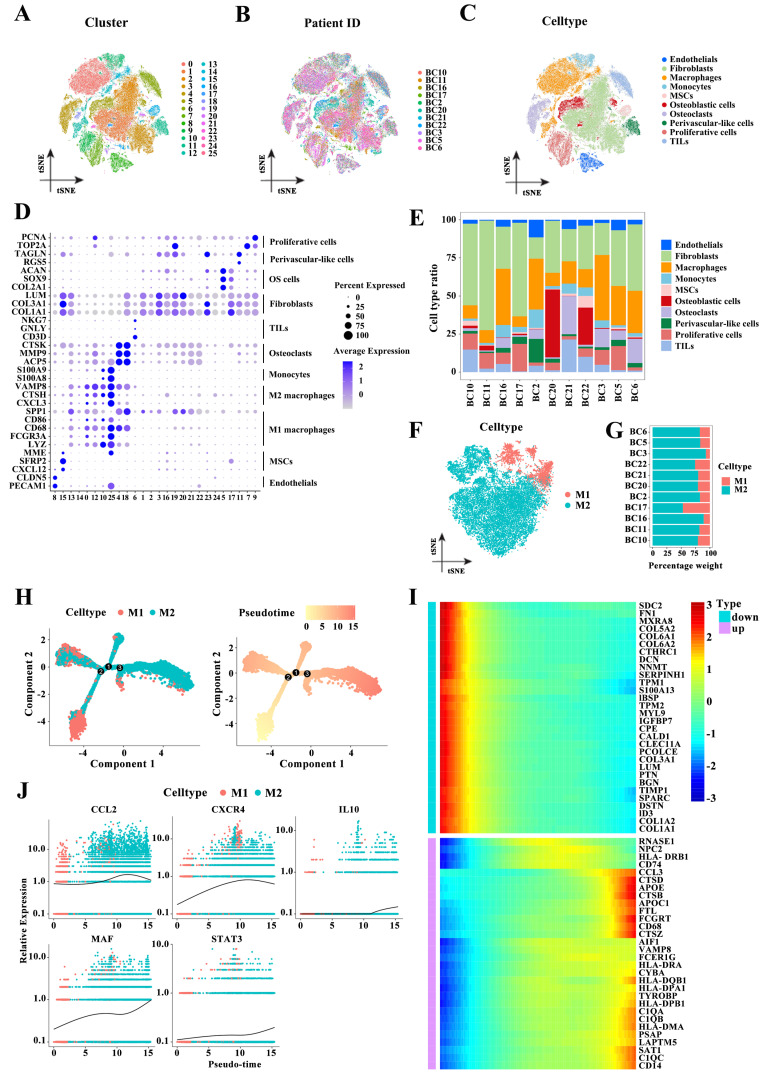
M2 macrophage landscape in the osteosarcoma microenvironment. (A-C) T-distributed stochastic neighbor embedding (t-SNE) plot of 109,747 cells from the GEO dataset GSE152048, colored based on cell clusters (A), patients (B), and cell types (C). (D) Expression levels of marker genes of 11 cell types in 26 cell clusters. (E) The proportion of 10 cell subpopulations in each patient. (F) t-SNE plot of macrophages colored based on macrophage subtypes. (G) The relative proportion of M1 and M2 macrophage in each patient. (H) Differentiation trajectory of macrophage cells colored based on macrophage subtypes or the pseudotime. (I) The heatmap for the top 30 highly expressed genes in M1- or M2 macrophages. (J) The pseudo-time trajectory pattern of the M2 polarization genes CCL2, CXCR4, IL10, MAF, and STAT3.

To describe the macrophage landscape in osteosarcoma, we first retrieved the scRNA-seq dataset of patients with osteosarcoma from the GEO database (GSE152048). After quantity control, we retained 109,747 cells of 11 osteosarcoma samples. We then reduced the dimensionality of the data by performing a principal component analysis on the 3000 most variable genes. The 26 cell clusters were visualized using the t-SNE algorithm (Figure 1A). Figure 1B shows the cell distribution of 11 osteosarcoma patients in the identified clusters. Based on the expression of known marker genes, these cell clusters were annotated as endothelial cells, fibroblasts, monocytes, mesenchymal stem cells (MSCs), osteoblastic cells, osteoclasts perivascular-like cells, proliferative cells, tumor-infiltrating lymphocytes (TILs), and macrophages (Figure 1C-D). Compared to other immune cell types, macrophages were a highly abundant cell type (Figure 1E). We next reclustered the macrophages and annotated these cells as M1 and M2 macrophages based on macrophage subtype marker genes (Figure 1F). M2 macrophages account for a high proportion in almost all the samples (Figure 1G). Diferentiation trajectory analysis of macrophages showed the transformation of M1 to M2 macrophages during osteosarcoma progression (Figure 1H), which was further supported by analyzing the relationship between the expression levels of the top 30 M1 and M2 macrophages or M2 polarization genes such as CCL2, CXCR4, IL10, MAF, and STAT3 and tumor progression (Figure 1I-J). Throughout, macrophages were the main immune cell type in the osteosarcoma microenvironment, and the high proportion of M2 macrophages may be caused by the transformation of macrophages M1 to M2.

### Cell-cell interactions in TME

**Figure 2:**
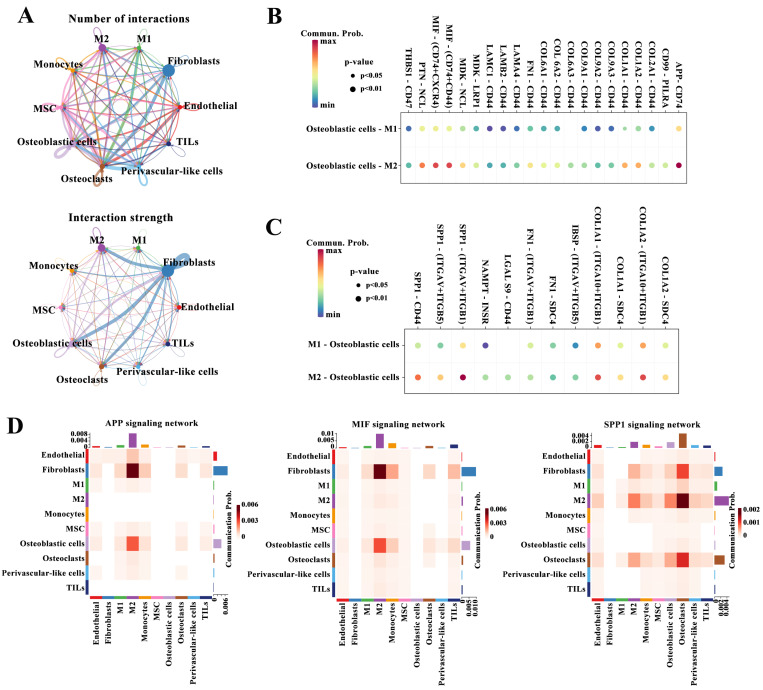
Cell-cell interactions. (A) Cell-cell interactions between different cell types. The width of the lines indicates the number (upper panel) or strength (down panel) of interactions. (B-C) The interactions between osteoblastic cells and M1 or M2 macrophages based on the expression of ligands from osteoblastic cells and receptors from M2 or M1 macrophages (B) or the expression of ligands from M2 or M1 macrophages and receptors from osteoblastic cells (C). (D) Heatmaps of the APP, MIF, and SPP1 signaling pathways between 10 populations.

Next, we performed cell communication analysis to explore the interactions between cell types. The numbers and strength of interactions among the identified cell types are shown in Figure 1A. The number of interactions between M2 and M1 macrophages and osteoblastic cells was similar, but the intensity of interactions between the former and osteoblasts was significantly higher than that between the latter and osteoblastic cells. The enhanced connections between M2 macrophages and osteoblastic cells were confirmed by detecting the interactions of ligands from osteoblastic cells with receptors from M2 or M1 macrophages such as APP-CD74, MIF-(CD74+CD44), and PTN-NCL (Figure 2B), or the interactions of ligands from M2 or M1 macrophages with receptors from osteoblastic cells such as SPP1-CD44, SPP1-(ITGAV+ITGB1), and COL1A2-SDC4 (Figure 2C). Figure 2D shows heatmaps of tumor progression-related pathways such as the amyloid-beta precursor protein (APP), migration inhibitory factor (MIF), and secreted phosphoprotein 1 (SPP1) signaling pathways between different cell types. These pathways were highly active between M2 macrophages and osteoblastic cells compared with the activity of the pathways between M1 macrophages and osteoblastic cells. Accordingly, M2 macrophages could communicate with osteoblastic cells through the APP, MIF and SPP1 signaling pathways, and these signals mediated by M2 macrophages may synergistically promote the malignant progression of tumors.

### Construction of prognostic model based on the M2 macrophage marker genes

**Figure 3:**
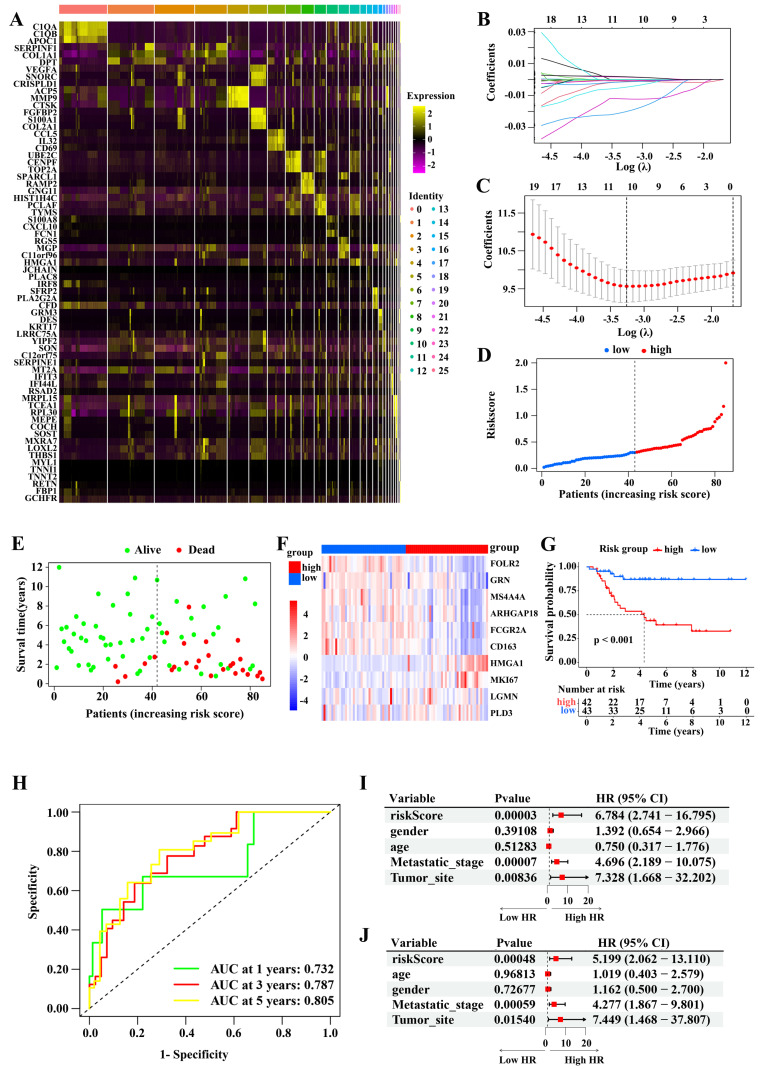
Construction of the prognostic model. (A) Heatmap of the three most expressed gene in each identified cell cluster. (B-C) LASSO regression analysis. (D-E) The distribution of risk score (D) and survival status (E) of patients from TCGA-osteosarcoma cohort. (F) Heatmap of the expression of the 10 gene in high- and low-risk subgroups. (G) The difference in the survival between high- and low-risk subgroups (Log-rank test). (H) The 1-, 3-, and 5-year ROC curves of the risk model. (I-J Relationships between risk score and overall survival times based on the univariable (I) and multivariable Cox regression analysis (J).

To identify M2 macrophage marker genes, we analyzed the differences in the gene expression between the 26 cell clusters, and determined 189 genes, namely osteosarcoma-related M2 macrophage marker genes. Figure 3A displays the expression profiles of the top three DEGs in each cell cluster. Using a univariate Cox regression analysis, we identified M2 macrophage marker genes clearly correlated with outcomes. To remove redundant genes, we performed a LASSO regression analysis (Figure 3B-C). From these, we screened out 10 genes (FOLR2, GRN, MS4A4A, ARHGAP18, FCGR2A, CD163, HMGA1, MKI67, LGMN, and PLD3). The constructed risk model had the following parameters: Risk score: = (-0.0010 × FOLR2 expression) + (-0.0006 × GRN expression) + (-0.0122 × MS4A4A expression) + (-0.0200 ×ARHGAP18 expression) + (-0.0048 × FCGR2A expression) + (-0.0013 × CD163 expression) + (0.0015 ×HMGA1 expression) + (0.0017 × MKI67 expression) + (-0.0001 × LGMN expression) + (-0.0015 × PLD3 expression). Base on the median risk score value (0.302), patients were divided into high- and low-score subgroups. The correlation between risk score and clinical characteristics in osteosarcoma patients from the TCGA-osteosarcoma cohort is shown in Table 1. Figure 3D-E displays the distribution of patients' risk scores or survival status, and indicates that most of the patients who did not survive belonged to the high-score subgroup. Figure 3F displays an expression heatmap of 10 genes in the high- and low-risk subgroups. The Kaplan-Meier analysis revealed that low-risk patients survived markedly longer than high-risk patients (Figure 3G). The area under the curves (AUCs) at 1, 3, and 5 years were 0.732, 0.787, and 0.805, respectively (Figure 3H). Moreover, the univariate and multivariate COX regression analysis demonstrated that the risk score was an independent indicator (Figure 3I-J).

### Validation of the ten-gene prognostic signature

**Figure 4:**
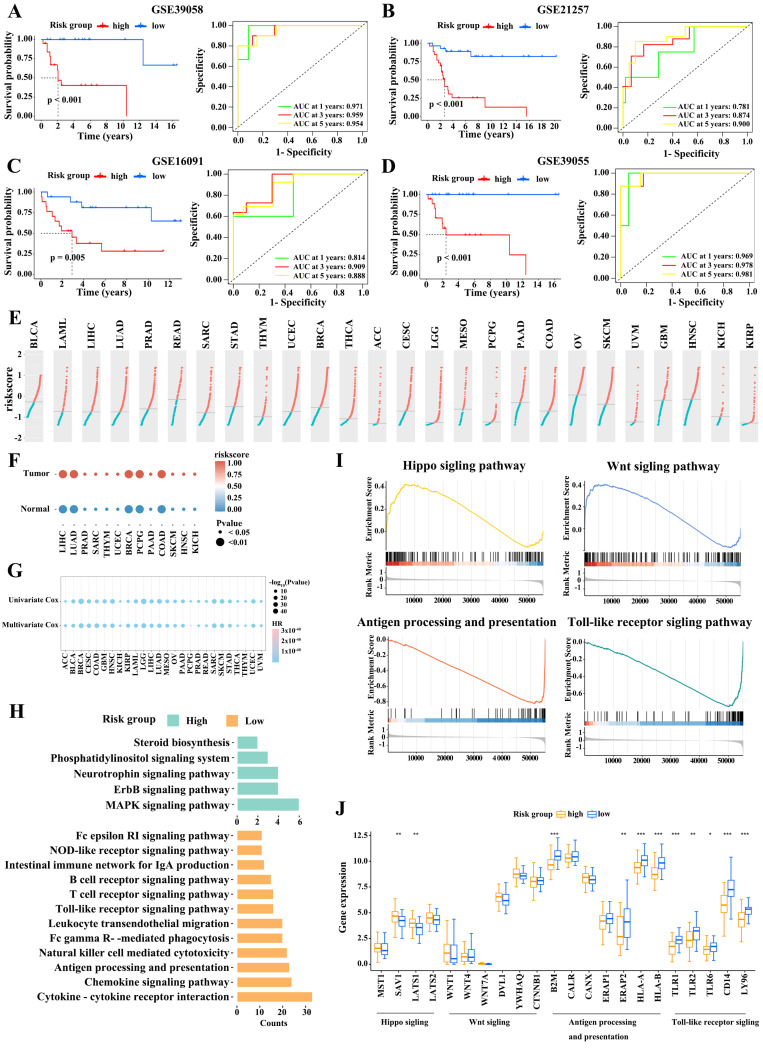
Validation of the risk model. (A-D) Survival comparison in the high- and low-risk subgroups (Log-rank test) and the 1-, 3-, and 5-year ROC curves of the risk model in GSE39058 (A), GSE21257 (B), GSE16091 (C), and GSE39055 (D) datasets (Log-rank test). (E) The risk scores of patients from 26 TCGA tumor types. (F) Evaluation of risk scores in tumor samples and adjacent normal samples in 13 TCGA tumor types. (G) Relationships between the risk score and overall survival times based on the univariable and multivariable Cox regression analysis in 26 TCGA tumor types. (H) Kyoto Encyclopedia of Genes and Genomes pathways enrichment analysis in high- and low-risk subgroups. (I) Gene set enrichment analysis.

Next, we validated the robustness of M2 macrophage marker gene signature using four independent cohorts from the GEO database (GSE39058, GSE21257, GSE16091, and GSE39055). We stratified the patients with osteosarcoma based on risk score as described above. The association between risk score and clinical characteristics in osteosarcoma patients from the four cohort is shown in Table 2-5, respectively. In the four cohorts, low-risk patients had significantly better outcomes than high-risk patients (Figure 4A-D). The AUCs at 1, 3, and 5 years in these cohorts also indicated that the risk model had good predictive accuracy. Therefore, the M2 macrophage marker gene signature was a predictor of outcome in patients with osteosarcoma.

Moreover, we assessed 9,757 samples across 26 tumor types, including bladder urothelial carcinoma (BLCA), acute myeloid leukemia (LAML), liver hepatocellular carcinoma (LIHC), and lung adenocarcinoma (LUAD), from the TCGA dataset. Figure 4E displays the distribution of risk scores for these tumor types. We then assigned tumor samples to the high- and low-score subgroups using the median score for each tumor type as the cutoff value. Remarkably, tumor tissues had higher risk scores than their matched adjacent normal tissues (Figure 4F). Univariate and multivariate survival analyses then revealed that a high risk score was consistently linked to poor outcomes in almost all tumor types; this was notably the case in LUAD, pancreatic adenocarcinoma (PAAD), mesothelioma (MESO), and uveal melanoma (UVM; Figure 4G). Accordingly, the M2 macrophage marker gene signature classification allowed to reliably predict cancer outcomes.

### Pathway enrichment analysis

To explore the potential mechanism contributing to the difference in outcome between with high- and low-risk patients, we performed a KEGG analysis. We found that immune-related pathways (such as antigen processing and presentation, T cell receptor signaling pathway, B cell cell receptor signaling pathway, Natural killer cell mediated cytotoxicity, and cytokine receptor interaction signaling pathway) were prominently enriched in the low-risk subgroup, while, in high-risk subgroup, pathways related to tumor formation and progression, were enriched (Figure 4H). A GSEA analysis confirmed these observations (Figure 4I).

### Risk score correlates with tumor immune microenvironment

**Figure 5:**
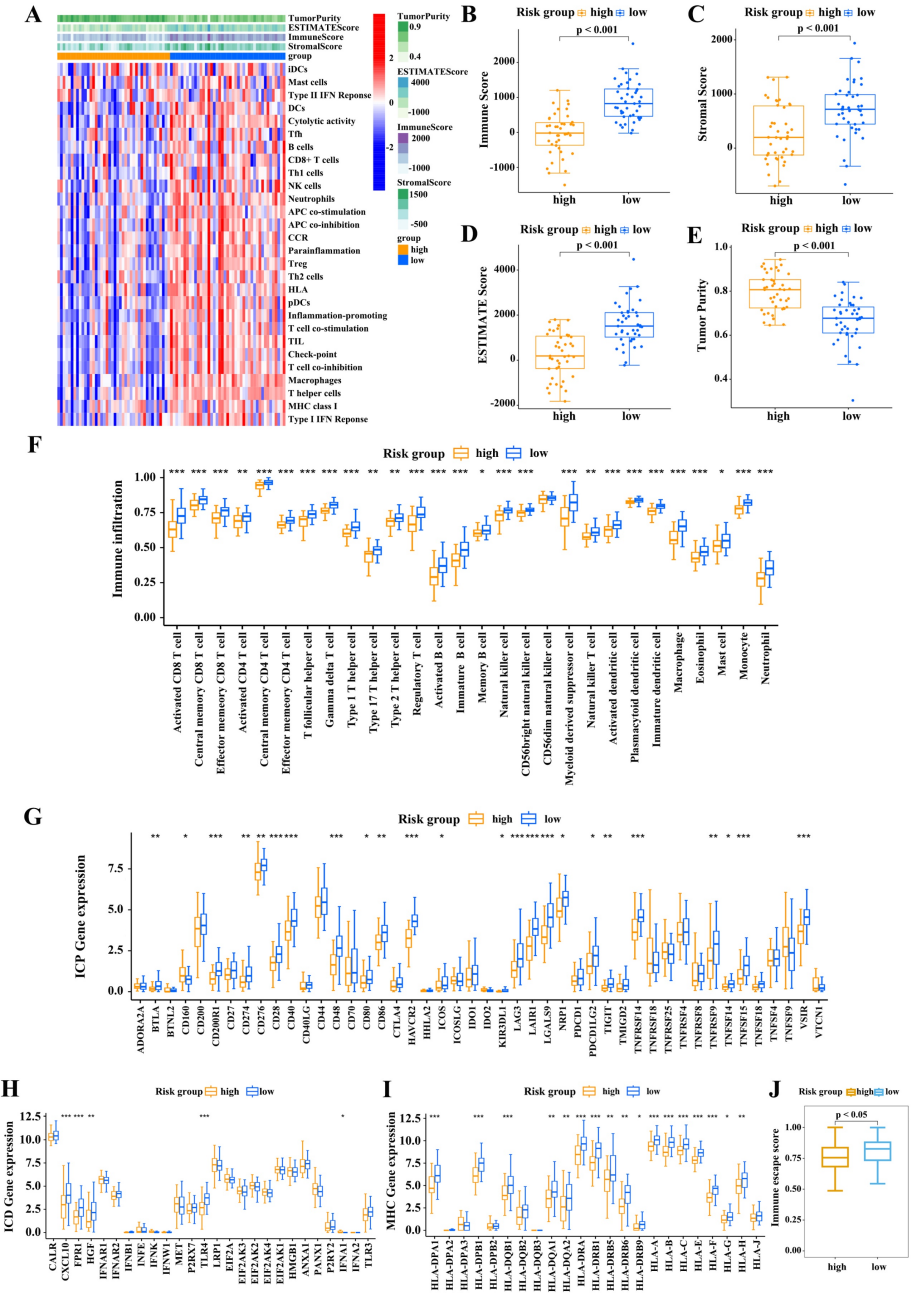
Associations between risk score and immune cell infiltration. (A) Heatmap of the infiltrated immune cell types in high- and low-risk subgroups. Immune score (B), stromal score (C), ESTIMATEscore (D), and tumor purity (E) in the two subgroups. (F) Infiltrated immune cell types in the two subgroups. (G-I) Differences in the levels of ICPs (G), ICD modulators (H), and MHC molecules (I) between high- and low-risk subgroups. (Wilcoxon test; *P < 0.05; **P < 0.01; ***P < 0.001).

We then assessed the relationship between risk score and the tumor immune microenvironment. The heatmap (Figure 5A) shows that immune-associated functions were more active in the low-risk subgroup than in the high-risk subgroup. We then calculated the immune score, stromal score, ESTIMATE score, and tumor purity of the two risk score subgroups by applying the ESTIMATE algorithm. As shown in Figure 5B-E, the low-score subgroup had significantly higher scores than high-risk subgroup. Next we analyzed the infiltration levels of the 28 immune cell types in these two subgroups using CIBERSORT algorithm. Low-risk patients clearly displayed high infiltration levels of almost all types of immune cells (Figure 5F). Accordingly, low-risk patients had a “hot” immune phenotype, while high-risk patients displayed an opposite immunologic feature, indicative of a “cold” immune phenotype.

We next explored the molecular features of the tumor immune microenvironment in the two risk subgroups. Among 46 ICPs, 23 were highly expressed in low-risk patients, including PD-L1, CTLA-4, and PDCD1 (Figure 5G). ICD is a key factor for improving cancer immunotherapy. Low-risk patients displayed enhanced expression of ICD regulators, such as CXCL10, FPR1, HGF, TLR4, and IFNA1 (Figure 5H). Furthermore, low-risk patients displayed high levels of HLA-related proteins (Figure 5I). These findings indicated that low-risk patients may benefit from immune checkpoint-based immunotherapy.

### Risk score predicting response to immunotherapy and drug sensitivity

**Figure 6:**
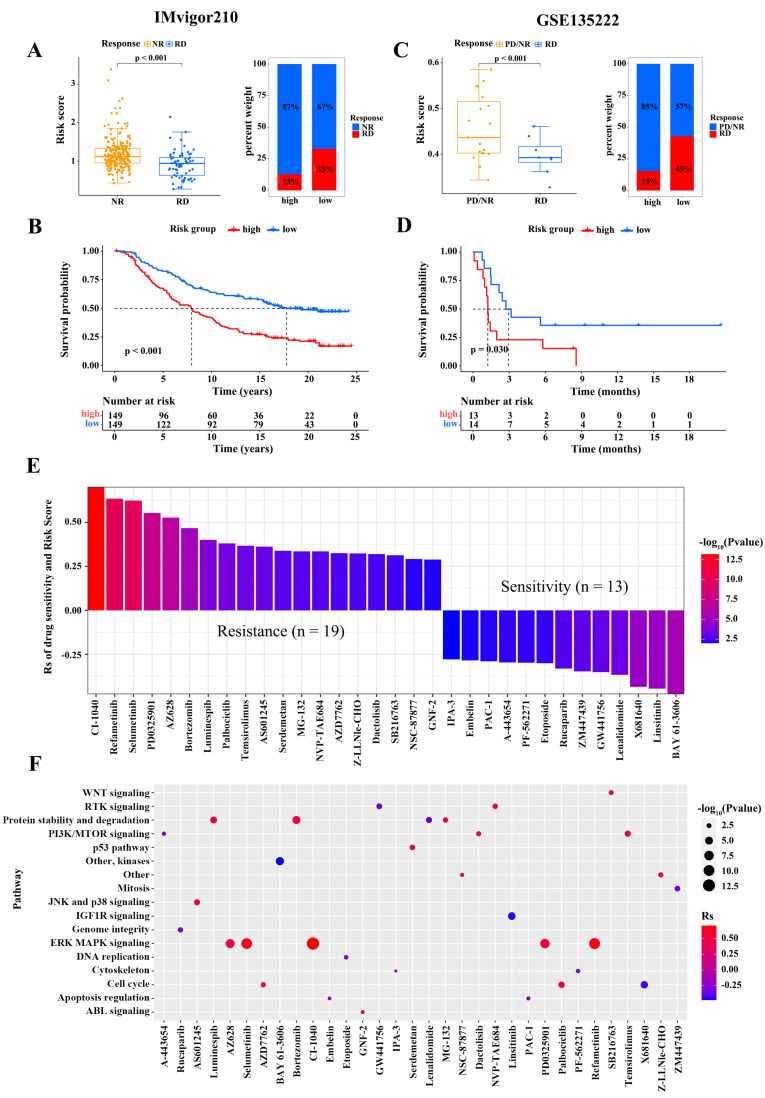
Relationship between risk score and immunotherapy response and drug sensitivity. (A) The difference in the risk scores between no response (NR) and complete response (RD) patients (Wilcoxon test) and the proportion of RD patients in the IMvigor210 cohort. (B) The difference in the survival between high- and low-risk subgroups in the IMvigor210 cohort (Log-rank test). (C) The difference in the risk scores between progressive disease (PD)/(NR) and RD patients (Wilcoxon test) and the proportion of RD patients in the GSE135222 cohort. (D) The difference in the survival between high- and low-risk subgroups in the GSE135222 cohort (Log-rank test). (E) Relationship of risk scores and drug sensitivity (Spearman analysis). (F) Signaling pathways targeted by drugs that were resistant (red) or sensitive (blue) to the risk score. (*P < 0.05; **P < 0.01; ***P < 0.001).

We assessed the capacity of risk score signature to the immunotherapy response in two independent cohorts, namely GSE135222 cohort (patients with advanced non-small cell lung carcinoma treated with anti-PD-1/PD-L1therapy) and IMvigor210 cohort (patients with advanced urothelial cancer treated with anti-PD-L1). In the IMvigor210 cohort, patients with RD had signficantly lower risk scores than patients with NR, and the percentage of patients with RD was higher in the low-risk subgroup (Figure 6A). The correlation between risk score and clinical characteristics in non-small cell lung cancer patients from the two cohort is shown in Table 6-7, respectively. The survival analysis revealed that the overall survival of low-score patients was remarkably better than that of high-score patients (Figure 6B). Similar results were observed in the GSE78220 cohort (Figure 6C-D). To further explore the potential pharmacotherapeutic value of risk scores, we analyzed the correlation between risk scores and the drug responses of cancer cell lines. A Spearman correlation analysis revealed that 32 drugs with a clear association between risk score and drug sensitivity (Figure 6E). Among them, 13 pairs exhibited that drug sensitivity was related to risk score, such as the Syk inhibitor BAY61.3606 (Rs = -0.48, p = 3.85 × 10^-6^), TNF-α inhibitor Lenalidomide (Rs = -0.37, p = 5.47 × 10^-14^), and insulin receptor (IR) and insulin-like growth factor-1 receptor (IGF-1R) inhibitor OSI.906 (Rs = -0.46, p = 1.92 × 10^-5^). Besides, the 19 pairs displayed that drug resistance was linked to risk score, such as the MEK inhibitor RDEA119 (Rs = 0.63, p = 6.43 × 10^-6^), Raf inhibitor AZ628 (Rs = 0.53, p = 2.11 × 10^-7^), and proteasome inhibitor Bortezomib (Rs = 0.47, p = 6.43 × 10^-6^). We subsequently evaluated the signaling pathways involved in these drug target genes, and found that the risk score was associated with drug-associated signaling pathways, such as WNT signaling pathway, PI3K/MTOR signaling pathway, JNK and p38 signaling pathway, IFG1R signaling pathway, ERK/MAPK signaling pathway, and DNA replication signaling pathway (Figure 6F). Taken together, these findings suggest that the risk score is a marker for assessing the response to immunotherapy and guide the choice of chemotherapeutic agents to treat patients with osteosarcoma.

## Discussion

Immunotherapy, using immune checkpoint inhibitors, has achieved positive results in the treatment of multiple tumor types, including osteosarcoma. It has become one of the most promising clinical strategies for treating cancer in recent years 18. However, identifying patients that could benefit from this treatment remains a huge challenge for osteosarcoma immunotherapy 19. Current research mainly focuses on bulk RNA-seq data. Osteosarcomas are heterogeneous bone tumors. Therefore, identification of effective biomarkers should take into account tumor heterogeneity. scRNA-seq technology, a powerful method to study tumor heterogeneity, has great potential for identifying prognostic and therapeutic markers. Here, we found that macrophages were the major immune cell type in the osteosarcoma microenvironment, and the high proportion of M2 macrophages might result from the transition of macrophages M1 to M2. M2 macrophages can communicate with osteoblastic cells by the APP, MIF, and SPP1 signaling pathways, promoting the malignant progression of tumors. These findings are supported by previous studies. For example, macrophage MIF promotes the progression of multiple tumor types by modulating the immune and inflammatory responses 20. Inhibition of the SPP1 pathway limits the malignant progression of melanoma 21. The APP signling pathway is associated with tumor metastasis 22. Next, we identified M2 macrophage marker genes in osteosarcoma using single-cell analysis, and built a prognostic model based on the expression profiles of 10 key M2 macrophage marker genes. CD163 and MS4A4A of these 10 genes have been proposed as M2 macrophage markers. We validated the outcome prediction ability of this model using four independent osteosarcoma cohorts from the GEO dataset. Patients with low risk scores showed significant enrichment of immune-related pathways and high abundance of immune cell infiltration. Moreover, low-risk patients treated with immunotherapy showed a significant survival advantage, suggesting that patients with low risk scores might benefit more from immune treatments based on immune checkpoint blockade.

The risk model was composed of the 10 M2 macrophage marker genes FOLR2, GRN, MS4A4A, ARHGAP18, FCGR2A, CD163, HMGA1, MKI67, LGMN, and PLD3. Previous studies have suggested that these marker genes were linked to cancer outcome or macrophage activity. For instance, FCGR2A encodes a member of the immunoglobulin Fc receptor proteins family that lies on the surface of macrophages and participates in phagocytosis and clearance of immune complexes 23. Its abnormal expression promotes osteosarcoma metastasis and is associated with poor outcomes in patients with osteosarcoma 24. HMGA1, a structural transcription factor, is highly expressed in tumor tissues; it modulates tumor growth and metastasis and is linked to unfavorable prognosis in patients with osteosarcoma 25. As a pleiotropic immunomodulatory molecule, CD163 not only stimulates the immune response but also promotes tumor cell proliferation, invasion and metastasis 26-27. MKI67, a prominent cancer marker, contributes to osteosarcoma progression by inducing cell proliferation 28. Therefore, the genes determined in our work might offer potential targets for further clinical research on osteosarcoma.

Our training and validation cohort results demonstrated that the risk model constructed in this study could be used to predict survival in patients with osteosarcoma and even performed well in pan-cancer outcome prediction. The excellent predictive power of this risk model motivated us to explore the underlying mechanisms. The poor outcome of high-risk patients might be ascribed to the activation of tumor progression-related pathways, such as ErbB signaling and the MAPK signaling pathway 29-30, while the functional enrichment of pathways associated with immune responses in low-risk patients contributed to their favorable outcomes. Accordingly, our study highlights the potential value of risk scoring in precision medicine against cancer.

Immunotherapy has been clinically approved to treat a multitude of cancers, but only a small proportion of patients have had meaningful treatment responses. Major effort have been made to determine markers allowing to predict response to cancer immunotherapy 31. In this study, we found a significant enrichment of infiltrated immune cells, including T and B cells, in the TME of low-risk tumors compared with how-risk tumors, implying that tumors with low risk scores are immunologically “hot tumors”, more sensitive to antitumor immunotherapy. Furthermore, immune checkpoint molecules serve as potential targets for cancer immunotherapy 32. Low-risk patients expressed high levels of immune checkpoint-related factors, indicating that these patients may reap more benefits from immunotherapy than high-risk patients. ICD activates adaptive immune response. Antigen presentation by MHC molecules is critical for adaptive immunity 33. A high expression of ICD regulators and HMC molecules in low-risk patients suggested that low-risk tumors had enhanced immunogenicity, and so were sensitive to immunotherapy. We verified this hypothesis using two independent immunotherapy datasets. Taken together, these results show that the risk model based on the M2 macrophage marker genes could be used as a marker for predicting the effects of immunotherapy, and patients with low-risk scores might potentially benefit more from the immunotherapy.

We also evaluated the ability of the risk model to predict pharmacotherapeutic effects. The risk score was sensitive to 13 anticancer drugs and it was resistance to 19 anticancer drugs, offering a reference for drug selection in osteosarcoma therapy. Previous studies have also shown that these drugs have positive effects in some patients with osteosarcoma 3. Moreover, the risk score was correlated with resistance to drugs targeting signaling pathways, such as the WNT, ERK/MAPK, and PI3K/mTOR signaling pathways, and with sensitivity to drugs targeting mitosis, DNA replication, and apoptosis regulation signaling. We, therefore, speculated that high-risk patients would response to drugs targeting these pathways, rather than to drugs targeting the WNT, ERK/MAPK, or PI3K/mTOR pathways. The risk model could serve as a predictor to assess the clinical outcome of drug therapies.

There were several limitations in our study. Although we have demonstrated that M2 macrophage marker gene signature can predict drug sensitivity, survival, and immunotherapy response, the molecular mechanism of interaction between macrophages and osteoblasts in osteosarcoma progression was still needed to be further confirmed.

## Conclusions

In summary, macrophages were the major immune cell type in the osteosarcoma microenvironment, and the high proportion of M2 macrophages might result from the transition of macrophages M1 to M2. M2 macrophages communicated with osteoblasts by the APP, MIF, and SPP1 signaling pathways, facilitating osteosarcoma development. We further constructed a ten-gene signature based on the identified M2 macrophage marker genes. The signature allowed to predict drug sensitivity, survival, and immunotherapy response. High-risk patients would response to drugs targeting mitosis, DNA replication, and apoptosis regulation signaling pathways. Our study provides new insights into the role of M2 macrophage marker genes, and contributed to optimize personalized prognosis and even to improve the outcome of immunotherapy for osteosarcoma patients. This study also offers provides a method for identifying effective biomarkers based on integrated analysis of single-cell and bulk RNA sequencing.

## Supplementary Material

Supplementary tables.

## Figures and Tables

**Table 1:** Correlation between risk score and clinical characteristics in osteosarcoma patients from the TCGA-osteosarcoma cohort.

Characteristic	Risk score (high)	Risk score (low)	*P* value
Number of cases (n)	42	42	
Gender, n (%)			0.66
Female	17 (40.5)	20 (47.6)	
Male	25 (59.5)	22 (52.4)	
Age, n (%)			0.247
<18	25 (59.5)	31 (73.8)	
>=18	17 (40.5)	11 (26.2)	
Metastatic stage, n (%)			0.044
Metastatic	15 (35.7)	6 (14.3)	
Non-metastatic	27 (64.3)	36 (85.7)	
Tumor site, n (%)			0.358
Arm/Hand	3 (7.1)	3 (7.1)	
Leg/Foot	37 (88.1)	39 (92.9)	
Pelvis	2 (4.8)	0 (0.0)	

**Table 2:** Correlation between risk score and clinical characteristics in osteosarcoma patients from the GSE16091 cohort.

Characteristic	Risk score (high)	Risk score (low)	*P* value
Number of cases (n)	17	17	
Age, n (%)			0.09
<18	11 (64.7)	16 (94.1)	
≥18	6 (35.3)	1 (5.9)	
Gender, n (%)			1
Female	6 (40.0)	7 (46.7)	
Male	9 (60.0)	8 (53.3)	
Tumor site, n (%)			0.474
Arm	1 (5.9)	0 (0.0)	
Distal femur	3 (17.6)	6 (35.3)	
Ethmoid sinus	0 (0.0)	1 (5.9)	
Femur	1 (5.9)	2 (11.8)	
Femur/tibia	1 (5.9)	0 (0.0)	
Humerus	2 (11.8)	2 (11.8)	
Left mandible	0 (0.0)	1 (5.9)	
LNS	3 (17.6)	0 (0.0)	
Lt proximal humerus	0 (0.0)	1 (5.9)	
Parietooccipital area	1 (5.9)	0 (0.0)	
Proximal femur	2 (11.8)	0 (0.0)	
Proximal humerus	1 (5.9)	1 (5.9)	
Proximal tibia	1 (5.9)	2 (11.8)	
Tibia	1 (5.9)	1 (5.9)	

**Table 3:** Correlation between risk score and clinical characteristics in osteosarcoma patients from the GSE21257 cohort.

Characteristic	Risk score (high)	Risk score (low)	*P* value
Number of cases (n)	26	27	
Age, n (%)			0.297
<18	19 (73.1)	15 (55.6)	
≥18	7 (26.9)	12 (44.4)	
Gender, n (%)			0.297
Female	7 (26.9)	12 (44.4)	
Male	19 (3.1)	15 (55.6)	
Tumor site, n (%)			0.339
Diaphysis of left femur	0 (0.0)	1 (3.7)	
Distal femur	1 (3.8)	0 (0.0)	
Femur	2 (7.7)	3 (11.1)	
Humerus	2 (7.7)	2 (7.4)	
Left distal femur	6 ( 23.1)	5 (18.5)	
Left femur	1 (3.8)	0 (0.0)	
Left proximal femur	0 (0.0)	1 (3.7)	
Left proximal fibula	0 (0.0)	1 (3.7)	
Left proximal humerus	2 (7.7)	0 (0.0)	
Left proximal tibia	3 (11.5)	4 (14.8)	
Right distal femur	1 (3.8)	5 (18.5)	
Right distal tibia	0 (0.0)	1 (3.7)	
Right humerus	1 (3.8)	0 (0.0)	
Right proximal femur	1 (3.8)	0 (0.0)	
Right proximal fibula	1 (3.8)	0 (0.0)	
Right proximal humerus	0 (0.0)	1 (3.7)	
Right proximal tibia	4 (15.4)	1 (3.7)	
Tibia	0 (0.0)	2 (7.4)	
Grade, n (%)			0.345
Ⅰ	8 (38.1)	5 (19.2)	
Ⅱ	6 (28.6)	10 (38.5)	
Ⅲ	4 (19.0)	9 (34.6)	
Ⅳ	3 (14.3)	2 (7.7)	
Metastases, n (%)			<0.001
Metastases	24 (92.3)	10 (37.0)	
No metastases	2 (7.7)	17 (63.0)	

**Table 4:** Correlation between risk score and clinical characteristics in osteosarcoma patients from the GSE39055 cohort.

Characteristic	Risk score (high)	Risk score (low)	*P* value
Number of cases (n)	18	18	
Age, n (%)			1
<18	16 (88.9)	16 (88.9)	
≥18	2 (11.1)	2 (11.1)	
Gender, n (%)			1
Female	8 (44.4)	9 (50.0)	
Male	10 (55.6)	9 (50.0)	

**Table 5:** Correlation between risk score and clinical characteristics in osteosarcoma patients from the GSE39058 cohort.

Characteristic	Risk score (high)	Risk score (low)	*P* value
Number of cases (n)	20	21	
Age, n (%)			1
<18	17 (85.0)	18 (85.7)	
≥18	3 (15.0)	3 (14.3)	
Gender, n (%)			1
Female	10 (50.0)	10 (47.6)	
Male	10 (50.0)	11 (52.4)	

**Table 6:** Correlation between risk score and clinical characteristics in non-small cell lung cancer patients from the GSE135222 cohort.

Characteristic	Risk score (high)	Risk score (low)	*P* value
Number of cases (n)	13	14	
Age, n (%)			0.322
<65	9 (69.2)	6 (42.9)	
≥65	4 (30.8)	8 (57.1)	
Gender, n (%)			1
Female	2 (15.4)	3 (21.4)	
Male	11 (84.6)	11 (78.6)	
Response, n (%)			0.254
PD/NR	11 (84.6)	8 (57.1)	
RD	2 (15.4)	6 (42.9)	

**Table 7:** Correlation between risk score and clinical characteristics in bladder cancer patients from the IMvigor210 cohort.

Characteristic	Risk score (high)	Risk score (low)	*P* value
Number of cases (n)	149	149	
Response, n (%)			<0.001
NR	130 (87.2)	100 (67.1)	
RD	19 (12.8)	49 (32.9)	
Stage, n (%)			0.04
Ⅰ	61 (40.9)	46 (30.9)	
Ⅱ	27 (18.1)	48 (32.2)	
Ⅲ	31 (20.8)	29 (19.5)	
Ⅳ	30 (20.1)	26 (17.4)	
