# Supplementary material for: Identification of M2 macrophage markers for predicting outcome and therapeutic response in osteosarcoma: Integrated analysis of single-cell and bulk RNA-sequencing
Source: J Cancer. 2025 Feb 28;16(6):1873–87. doi: 10.7150/jca.104855 (PMC11905420; doi:10.7150/jca.104855)
Supplement: Supplementary file 1 — Supplementary tables. [file jcav16p1873s1.pdf]

Table S1 Clinical characteristics in osteosarcoma patients from the GSE152048 cohort.

Sample	Type	Location	Preoperative chemotherapy	Size (cm)	Necrosis rate	Ki67+
BC2	Primary	Femur	4 times (MTX, AP, IFO, MTX)	5.5*5*3	＜90%	50%
BC3	Primary	Tibia	6 times (MTX, AP, MTX, AP, MTX, MTX)	8*6*6	＜90%	70%
BC5	Primary	Femur	3 times (MTX, IFO, AP)	8*7.5*6	⩾90%	80%
BC6	Primary	Ulna	3 times (MTX, IFO, AP)	7*7*4	⩾90%	15%
BC10	Metastasis	Femur	2 times (GT, GT)	3.5*3*2	＜90%	8%
BC11	Recurrent	Femur	3 times (GT, GT, GT)	20*11*10	＜90%	30%
BC16	Primary	Tibia	4 times (IFO, AP, MTX, MTX)	6*4*2.5	＜90%	40%
BC17	Metastasis	Tibia	3 times (GT, GT, GT)	2*2*1.5	＜90%	40%
BC20	Recurrent	Femur	4 times (AP, IE, AP, IE)	10*8*5	＜90%	50%
BC21	Primary	Femur	4 times (MTX, AP, IFO, AP)	5*4*1	＜90%	10%
BC22	Primary	Femur	4 times (MTX, AP, IFO, MTX)	18*15*12	＜90%	20%
BC22	Primary	Femur	4 times (MTX, AP, IFO, MTX)	18*15*12	＜90%	20%

Abbreviations: MTX, Methotrexate; AP, Doxorubicin + Cisplatin; IE, IFO + VP-16; IFO, Ifosfamide; GT, Gemcitabine + Docetaxel; VP-16, Etoposide.

Table S2 Basic information of public datasets.

Dataset	Data Type	Sample Type	Organism	Description of samples in this study	Figures Displayed in	PubMed ID
GSE152048	scRNA-seq	osteosarcoma	Homo sapiens	the scRNA-seq anakysis of 11 osteosarcoma tissues based on the 10X Genomics platform	Figure 1,2	PMID:33303760
GSE39058	RNA-seq	osteosarcoma	Homo sapiens	47 patients in human osteosarcoma	Figure 4	PMID:23339462
GSE21257	RNA-seq	osteosarcoma	Homo sapiens	Pre-chemotherapy biopsies of 53 osteosarcoma patients	Figure 4	PMID:21372215
GSE16091	RNA-seq	osteosarcoma	Homo sapiens	34 patients in human osteosarcoma	Figure 4	PMID:20028558
GSE39055	RNA-seq	osteosarcoma	Homo sapiens	37 patients in human osteosarcoma	Figure 4	PMID:38946948
TCGA	RNA-seq	33 cancer types	Homo sapiens	matched tumor samples spanning 33 cancer types	Figure 3,4,5,6	PMID:24071849
IMvigor210	RNA-seq	BLCA	Homo sapiens	298 patients with anti-PD-1 therapy	Figure 6	PMID:32416780
GSE135222	RNA-seq	NSCLC	Homo sapiens	27 NSCLC patients treated anti-PD-1	Figure 6	PMID:31537801
GSE152048	scRNA-seq	osteosarcoma	Homo sapiens	the scRNA-seq anakysis of 11 osteosarcoma tissues based on the 10X Genomics platform	Figure 1,2	PMID:33303760
